# PD-L1/*CD274* and miR-155*/MIR155HG* Genetic Variants as Prognostic and Risk Biomarkers in Diffuse Large B-Cell Lymphoma

**DOI:** 10.3390/cancers18030469

**Published:** 2026-01-30

**Authors:** Marija Elez, Debora Misic, Gordana Velikic, Jelena Karajovic, Lavinika Atanaskovic, Gordana Supic

**Affiliations:** 1 Clinic of Hematology, Military Medical Academy, 11000 Belgrade, Serbia; drmarija.elez@gmail.com (M.E.); lavinika74@yahoo.com (L.A.); 2 Medical Faculty of Military Medical Academy, University of Defense, 11000 Belgrade, Serbia; karajovicjelena@gmail.com; 3 Institute for Medical Research, Military Medical Academy, 11040 Belgrade, Serbia; deborastefik@gmail.com; 4 Hajim School of Engineering, University of Rochester, Rochester, NY 14627, USA; gordana.velikic@gmail.com; 5 Clinic of Endocrinology, Military Medical Academy, 11000 Belgrade, Serbia

**Keywords:** diffuse large B-cell lymphoma, programmed cell-death protein ligand 1, *CD274*, miR-155, *MIR155HG*, genetic variants, prognosis, survival, susceptibility

## Abstract

**Background/Objectives**: Diffuse large B-cell lymphoma (DLBCL) is an aggressive and heterogeneous malignancy, for which predicting clinical outcomes remains challenging. Although immune-checkpoint pathways are known to influence tumor biology, the impact of their germline variants on DLBCL susceptibility and prognosis has not been fully elucidated. **Methods**: Variants in PD-L1 gene *CD274* (rs4143815, rs822336), and miR-155 gene *MIR155HG* (rs767649, rs1893650), assessed by TaqMan assays in 99 DLBCL patients and 113 age- and sex-matched healthy controls, were associated with clinicopathological features, treatment response, overall survival (OS), relapse-free survival (RFS), and disease susceptibility. **Results**: The PD-L1 variant rs822336 was significantly associated with relapse status (*p* = 0.005) and RFS (*p* = 0.008), with the wild-type GG genotype showing the poorest RFS that remained independent in the multivariate Cox analysis (HR = 2.387, *p* = 0.003). Conversely, rs4143815 showed a nominal association with treatment resistance (*p* = 0.026), while patients carrying the GG genotype had worse OS (*p* = 0.006). In susceptibility analyses, miR-155 variant rs767649 showed a nominal association with DLBCL risk, with the rare AA genotype showing an increased risk of DLBCL (OR = 5.234, *p* = 0.045), which did not remain significant after Bonferroni correction. **Conclusions**: In a hypothesis-generating manner, these findings suggest that PD-L1 genetic variants may predominantly influence disease progression and outcomes, while miR-155 variation may contribute to DLBCL susceptibility. These findings highlight germline immunogenetic variants as stable, treatment-independent markers that may inform future studies on risk stratification and prognosis in DLBCL.

## Simple Summary

Diffuse large B-cell lymphoma (DLBCL) is an aggressive cancer with highly variable outcomes. In this study, we explored whether DLBCL behavior is influenced by inherited genetic changes in two immune-regulatory genes: the gene coding for the immune-checkpoint molecule PD-L1, which cancer cells use to evade anti-tumor immunity, and miR-155, involved in the regulation of inflammation and immune signaling. A total of 99 DLBCL patients and 113 healthy individuals were included in this study. We observed that specific variants in the PD-L1-encoding gene were associated with treatment response, relapse, and survival, depending on the genetic pattern. A variation in miR-155 was associated with an increased DLBCL risk. These hypothesis-generating results suggest that immune-related germline variants may contribute to inter-individual differences in DLBCL risk and outcome and warrant further investigation in larger, independent cohorts.

## 1. Introduction

Diffuse large B-cell lymphoma (DLBCL) represents the most frequent subtype of B-cell non-Hodgkin lymphoma (B-NHL). This aggressive and heterogeneous disease comprises different histopathological and molecular subtypes with distinct clinical presentation, genetic background, prognosis, and response to therapy [1,2,3]. Despite significant therapeutic progress over the past decades, particularly the incorporation of rituximab in combination with cyclophosphamide, doxorubicin, vincristine, and prednisone (R-CHOP), approximately 30–50% of patients still experience treatment failure, relapse, or refractory disease [4].

Molecular profiling has refined DLBCL classification into germinal-center B-cell-like (GCB) subtype, activated B-cell-like (ABC) subtype, and unclassified cases. These subtypes arise from different stages of lymphoid cell differentiation (cell of origin) and exhibit distinct biological behavior and clinical outcomes, with the ABC-DLBCL subtype generally associated with poorer prognosis compared to GCB-DLBCL [1,2,3]. Although the International Prognostic Index (IPI), which integrates age, stage, lactate dehydrogenase (LDH) level, extranodal involvement, and performance status, remains an essential clinical tool, substantial outcome variability still persists within IPI-matched groups [5]. This highlights the need for additional prognostic biomarkers that can refine patient stratification and guide personalized therapy.

The tumor microenvironment (TME) plays a central role in lymphoma biology. It represents a complex and dynamic network composed of malignant, immune, and stromal cells, extracellular-matrix components, soluble mediators such as cytokines, chemokines, and growth factors, and the vascular and lymphatic systems [6]. Within this microenvironment, effective antitumor immunity depends on the coordinated activity of cells involved in innate and adaptive immune responses. Dendritic cells (DCs), as professional antigen-presenting cells, capture and process tumor antigens and present them to naive T lymphocytes via major histocompatibility complex (MHC) molecules, thereby initiating activation of CD4^+^ helper T-cells (Th1 and Th2 subpopulations) and CD8^+^ cytotoxic T lymphocytes (CTLs). Once activated, CTLs recognize and directly destroy tumor cells, while Th1 cells further enhance the immune response by secreting interferon gamma (IFN-γ) and other pro-inflammatory cytokines [7].

To maintain immune homeostasis and prevent excessive tissue damage, inhibitory immune-checkpoint molecules, particularly programmed cell-death protein 1 (PD-1) and its ligands PD-L1 and PD-L2, attenuate T-cell activation. PD-1 (encoded by *PDCD1*) is expressed on activated T-cells, B-cells, and natural-killer (NK) cells, whereas PD-L1 (encoded by the cluster of differentiation 274 (*CD274*)) and PD-L2 (encoded by *CD273*) are expressed on both immune and tumor cells [8]. Their interaction transmits inhibitory signals that suppress T-cell proliferation and enhance regulatory T-cell (Treg) activity, ensuring peripheral tolerance. Under physiological conditions, PD-1/PD-L1 signaling attenuates T-cell-receptor (TCR) activity and limits proliferation and activation of effector cells and excessive inflammation, thereby avoiding immune-mediated tissue injury and preventing autoimmunity [7,9]. However, tumor cells frequently exploit this pathway by upregulating PD-L1, enabling escape from immune surveillance and CTLs. PD-1/PD-L1 interaction establishes an immunosuppressive niche within the TME that promotes tumor growth and immune escape [8]. In DLBCL, PD-L1 overexpression has been documented in both malignant B-cells and tumor-infiltrating immune cells, especially in ABC-DLBCL, correlating with aggressive behavior and poor prognosis [10,11]. PD-L1 is therefore a biologically relevant prognostic biomarker.

Furthermore, PD-L1 is a major target of immune-checkpoint inhibitors (ICIs) like nivolumab and pembrolizumab, which are frequently used in melanoma, head and neck cancers, and Hodgkin lymphoma [9]. Although immunotherapy has revolutionized cancer treatment, its efficacy in DLBCL remains limited and variable [12,13], emphasizing the need for predictors of immunotherapy response.

Non-coding RNAs (ncRNAs) have emerged as pivotal regulators of oncogenesis and immune modulation. MicroRNAs (miRNAs), a subset of small ncRNAs, approximately 22 nucleotides in length, regulate gene expression at the post-transcriptional level by promoting the degradation of mRNA or inhibiting translation and play a key role in both homeostasis and pathological conditions such as tumors [14]. Among these, miR-155 is one of the most extensively studied oncomiRs, a microRNA with oncogenic roles. Transcribed from its host gene *MIR155HG*, miR-155 promotes malignant transformation by targeting genes involved in apoptosis, differentiation, proliferation, and immune signaling [15]. Its induction by NF-κB and AP-1 connects inflammation to tumorigenesis, while its overexpression suppresses tumor-suppressor genes such as SOCS1, contributing to immune escape and therapy resistance [16]. Importantly, miR-155 has been implicated in post-transcriptional suppression of PD-L1 mRNA, reinforcing immunosuppression within the TME [17]. Dysregulation of the miR-155/*MIR155HG* axis has been associated with aggressive clinical behavior and unfavorable prognosis across multiple malignancies, including DLBCL [18].

Given the biological and clinical heterogeneity of DLBCL and the significant percentage of patients who experience relapse or refractory disease, identifying novel prognostic biomarkers is urgently needed. Although several single-nucleotide polymorphisms or variants (SNPs/SNVs) in PD-L1 coding gene have been shown to have functional relevance in solid tumors [9,19,20], data on their impact in lymphomas remain scarce and did not show association with survival or susceptibility in investigated populations [21,22]. Likewise, a germline variation in miR-155/*MIR155HG* may alter transcriptional activity or miRNA processing, potentially influencing immune regulation and tumor behavior [23]. These variants could therefore shape tumor susceptibility, therapeutic response, relapse, and survival.

We hypothesized that germline variation within this immune-regulatory axis could contribute to differences in therapy response, relapse risk, and survival, potentially useful for patient stratification and individualized management. Accordingly, the present study investigates functional variants within the PD-L1/*CD274* gene and miR-155/*MIR155HG* to evaluate their clinical relevance and prognostic potential in DLBCL. Our findings suggest that these immune-related variants may support improved risk stratification and contribute to precision-medicine approaches for patients with DLBCL.

## 2. Materials and Methods

### 2.1. Study Subjects

This study included 99 patients with newly diagnosed diffuse large B-cell lymphoma (DLBCL), treated at the Clinic of Hematology, Military Medical Academy (MMA), Belgrade, Serbia. All participants in both the DLBCL cohort and the control group were Caucasians of Serbian origin. The control group consisted of hospital-based, healthy, unrelated individuals without a history of malignant, autoimmune, chronic inflammatory diseases, or chronic infections. Exclusion criteria included prior malignancy and hematologic diseases, as assessed by clinical interviews.

Clinical and laboratory data in the DLBCL cohort were collected at diagnosis and during the follow-up period, including age, sex, stage, lactate dehydrogenase (LDH), erythrocyte sedimentation rate (ESR), Eastern Cooperative Oncology Group (ECOG) performance status, extranodal involvement, and cell-of-origin subtype (germinal-center B-cell-like (GCB), activated B-cell-like (ABC), or unclassified DLBCLs). Treatment response (complete response (CR), partial response (PR), and refractory disease), relapse status, autologous stem cell transplantation (ASCT), and International Prognostic Index (IPI) scores were recorded according to standard clinical criteria.

The study was approved by the Ethics Committee of the Military Medical Academy, Belgrade, Serbia (approval number 1494-6), and the Ethics Committee of the Medical Faculty of the Military Medical Academy, Belgrade, Serbia (approval number 52/2024). All procedures were conducted in accordance with institutional regulations and the ethical standards of the Declaration of Helsinki. Written informed consent was obtained from each patient before enrollment.

### 2.2. DNA Isolation and Genotyping

Peripheral blood collected at diagnosis was used for genomic DNA extraction, which was performed using the ExtractMe genomic DNA kit (Blirt, Gdańsk, Poland), following the manufacturer’s instructions. Four variants, PD-L1-encoding gene *CD274* (rs4143815 (C>G) and rs822336 (G>C)), and miR-155/*MIR155HG* (rs767649 (T>A) and rs1893650 (T>C)), were selected based on their putative regulatory roles in PD-L1 and miR-155 pathways. Genotyping was performed by allelic discrimination quantitative PCR (qPCR) using predesigned TaqMan SNP Genotyping Assays (Applied Biosystems, Foster City, CA, USA) on the QuantStudio 5 Real-Time PCR System. PD-L1-encoding gene *CD274* rs4143815 (assay ID: C__31941235_10) and rs822336 (assay ID: C__1348559_10), and miR-155/*MIR155HG* rs767649 (assay ID: C__2212229_10) and rs1893650 (assay ID: C__11728421_10).

All analyzed variants represent germline (inherited) SNVs. Family members were not included, and segregation analysis was not performed.

### 2.3. In Silico Bioinformatics Analysis

Functional annotation of the investigated polymorphisms was carried out using publicly available bioinformatics tools. HaploReg v4.2 and the Ensembl genome browser were used to examine regulatory elements and chromatin states, including enhancer and promoter histone marks. Population-level allele frequencies were compared with reference data from the NCBI ALFA database and Ensembl (Release 115, GRCh38). Potential regulatory activity was additionally assessed using RegulomeDB 2.2, which integrates transcription factor binding and ENCODE ChIP-seq datasets. For variants rs767649 and rs1893650 within the miR-155/*MIR155HG* locus, predicted target interactions and pathway enrichment were explored using TargetScanHuman v8.0 and miRDB v6.0.

### 2.4. Statistical Analysis

Statistical analyses were performed using SPSS Statistics version 20.0 (IBM, New York, NY, USA). Associations between genotypes and categorical clinical variables were examined using the chi-square test or Fisher’s exact test when appropriate. Genotype distributions were examined in relation to clinicopathological variables, including sex (male vs. female), age (≤60 vs. >60 years), disease stage (I/II vs. III/IV), ESR (<55.5 vs. ≥55.5 mm/h), LDH (≤250 vs. >250 U/L), DLBCL subtype (ABC, GCB, unclassified), ECOG performance status (<2 vs. ≥2), extranodal involvement, treatment response (CR, PR, refractory), relapse status, and ASCT. For the associations with the genotypes of the examined genes, IPI risk was divided into two categories: low (low/intermediate low, IPI Score 0–2) and high (intermediate high/high, IPI Score 3–5).

Overall survival (OS) was defined as the time from diagnosis to death from any cause or last follow-up, whereas recurrence-free survival (RFS) represented the interval between diagnosis and the first documented relapse or last follow-up. Survival analyses were performed using the Kaplan–Meier method. Differences between survival curves were assessed with the log-rank test. Univariate and multivariate Cox proportional hazards regressions were used to estimate hazard ratios (HRs) and 95% confidence intervals (CIs). For the Cox hazards models, continuous clinical variables were analyzed in their original continuous form rather than being dichotomized. The multivariate Cox regression analysis examined all the variables that were statistically significant in the univariate analysis, including those with *p* < 0.100, by stepwise variable selection via the forward LR (Likelihood Ratio) method.

Logistic regression analysis was performed to evaluate the association between each investigated polymorphism and the risk of developing DLBCL. The analysis compared genotype distributions between the DLBCL cohort and an age- and sex-matched control group, consisting of healthy individuals, Caucasians of the same Serbian origin as the DLBCL cohort, without any history of cancer, autoimmune, or chronic inflammatory diseases. Exclusion criteria included prior malignancy, hematologic disorders, chronic infection, immunosuppressive therapy, and a family history of lymphoma. Control status was confirmed through structured questionnaires, clinical exam, and review of medical history. Odds ratios (ORs) and 95% confidence intervals (CIs) were calculated using models adjusted for age and sex to account for potential confounding, as age and sex were the only cancer-relevant variables available for controls. Genetic effects were assessed under multiple inheritance models, including additive, dominant, recessive, over-dominant, and full genotypic models, to characterize the contribution of each genotype to disease susceptibility.

Power analyses were performed separately for case–control and survival endpoints. At the study design stage, sample size considerations were guided by the Genetic Power Calculator (GPC) [24], indicating that cohorts of approximately 100 cases and controls provide adequate power to detect moderate-to-large genetic effects for common variants under standard inheritance models. GPC was assessed to estimate detectable ORs under dominant and recessive inheritance models, taking into account allele frequency, disease prevalence, and sample size. In addition, power analyses for DLBCL susceptibility in case–control comparisons were assessed using G*Power (version 3.1), based on χ^2^ contingency tables, 2 × 3 genotype distributions, or 2 × 2 dominant/recessive models, taking into account the genotype grouping. For survival analyses, power was determined by Schoenfeld’s method, and minimal detectable HRs were estimated based on the number of observed events and genotype grouping, consistent with the Kaplan–Meier and Cox proportional hazards models. Calculations assumed 80% power and a two-sided significance level of α = 0.05.

Two-sided *p* < 0.05 values were considered nominally statistically significant. To account for multiple testing at the variant level, a conservative Bonferroni correction was applied for four analyzed variants (α = 0.05/4), resulting in a corrected significance threshold of α = 0.0125. Associations meeting this threshold were considered robust, whereas nominal *p*-values not surviving correction, including subgroup and alternative inheritance-model analyses, were interpreted as hypothesis-generating or exploratory.

## 3. Results

### 3.1. DLBCL Patients Characteristics

Detailed patient characteristics are given in Table 1. In summary, a total of 99 DLBCL patients were enrolled in the current study, including 63 males and 36 females, with an age distribution showing 59 patients ≤60 years and 40 patients >60 years. The majority of patients had advanced-stage disease (III/IV, 68/99; 68.7%), while 31/99 (31.3%) had early-stage disease (I/II). Elevated LDH (>250 U/L) was found in 50 patients, and higher ESR values in 40 patients. ECOG performance status ≥2 was observed in 45 patients, reflecting a clinically heterogeneous cohort. Regarding molecular subtype, 29 cases were classified as ABC, 23 as GCB, and 46 remained unclassified. Extranodal involvement was observed in 54 of 99 patients.

Based on IPI scoring, 65 patients were grouped into the low/low-intermediate risk category (IPI 0–2), while 34 patients fell into the high/intermediate-high category (IPI 3–5). Treatment outcomes showed that 73 patients achieved complete remission (CR), 10 achieved partial remission (PR), and 18 exhibited primary refractory disease. Seventeen patients underwent autologous stem-cell transplantation (ASCT) as part of their treatment strategy. During follow-up, 24 patients (24.2%) experienced relapse, while the remaining 75 remained relapse-free.

### 3.2. In Silico Functional Analysis of Candidate Genetic Variants

In silico analysis of the four analyzed variants, PD-L1 rs4143815, PD-L1 rs822336, miR-155 rs767649, and MIR155HG rs1893650, indicated that each genetic variant resides within transcriptionally active regulatory regions enriched for enhancer or promoter histone marks, Table 2.

The PD-L1 gene variants rs4143815 and rs822336 are located in distinct regulatory regions of the *CD274* gene, affecting a 3′UTR miRNA-binding site and the promoter region, respectively. *MIR155HG* genetic variants rs767649 and rs1893650 are localized in the enhancer region and intronic elements of *MIR155HG*, which regulate transcription of noncoding RNAs miR-155/*MIR155HG*.

Although all of the analyzed genetic variants’ CADD scores were low and GERP values indicated no or weak evolutionary conservation, the presence of active chromatin signatures, transcription-factor motif alterations, and previously reported associations with immune signaling supports the notion that these common polymorphisms may exert subtle but biologically relevant effects on PD-L1 or miR-155 expression. Overall, the regulatory context of these loci suggests a potential contribution to inter-individual variability in immune regulation and tumor behavior in DLBCL.

### 3.3. Genotype Distributions and Associations with Clinicopathological Features

Associations between genotype distributions and clinicopathological variables, including LDH (≤250 vs. >250 U/L), DLBCL subtype (ABC, GCB, unclassified), ECOG performance status (<2 vs. ≥2), extranodal involvement, treatment response (CR, PR, refractory), ASCT, and relapse status, are summarized in Table 3. IPI risk was classified into two categories: Low (Low/Intermediate low, IPI Score 0–2) or High (Intermediate high/High, IPI Score 3–5). Across all four investigated genetic variants, no significant associations were observed between genotype distribution and sex, age, DLBCL subtype, disease stage, ESR, LDH, ECOG performance status, extranodal involvement, IPI risk groups, or ASCT, as shown in Table 3.

A nominal association was observed between the PD-L1 gene variant rs4143815 and therapy response (*p* = 0.026). However, this association did not remain significant after Bonferroni correction for multiple testing (α = 0.0125) and should therefore be interpreted as exploratory. Among patients with a complete response, the majority were wild-type (wt) C-allele carriers of the CC genotype (34/73, 46.6%) or CG (31/73, 42.5%) genotypes, whereas only eight were GG carriers. In contrast, the majority of patients with refractory disease were variant G-allele carriers of the GG genotype (7/18, 38.9%) or CG genotype (7/18, 38.9%), while a lower number were CC carriers (4/18, 22.2%), demonstrating the higher prevalence of the variant rs4143815 G-allele among patients with poor treatment response.

In contrast, a significant association was observed between PD-L1 gene variant rs822336 and relapse status (*p* = 0.005), which persisted after Bonferroni correction, Table 3. Among relapse-free patients, the majority were the variant C-allele carriers, with the GC (37/75, 49.3%) or CC (23/75, 30.7%) genotypes, whereas only 15 individuals had the wt GG genotype (15/75, 20%). Conversely, relapse occurred most often in wt GG genotype carriers (13/28, 46.4%), compared with seven GC genotype (7/28, 25%) and CC carriers (4/28, 14.3%), indicating that variant C-allele genotypes (GC/CC) are protective, while wt GG is associated with an increased risk of relapse. No additional significant associations were found for the remaining investigated genetic variants in miR-155/*MIR155HG*.

### 3.4. Cox Regression Analysis

Univariate Cox regression analysis identified several clinical and genetic variables associated with OS and RFS, Table 4. Among clinical parameters, therapy response (HR = 3.906, *p* = 0.000001), IPI score (HR = 1.932, *p* = 0.0002), and relapse status (HR = 3.764, *p* = 0.0004) emerged as significantly associated with OS, indicating worse outcomes in patients with refractory disease, higher IPI scores, or disease relapse. ECOG performance status demonstrated a borderline trend of association with OS (*p* = 0.051) in our cohort. Among the genetic variants, PD-L1 rs4143815 was nominally associated with OS, where homozygous GG genotype carriers had reduced survival compared to reference CC genotype (HR = 1.927, *p* = 0.013). However, this association did not reach the Bonferroni-corrected threshold.

Regarding RFS, IPI score emerged as a significant predictor of shorter RFS (HR = 1.633, *p* = 0.012), while response to therapy showed a strong trend toward association with RFS (*p* = 0.055). PD-L1 rs822336 showed a significant association with RFS and revealed that the wt GG genotype increased the risk of recurrence compared to the reference variant CC genotype, which showed a protective effect (HR = 2.137, *p* = 0.011). The association between PD-L1 rs822336 and RFS remained significant after Bonferroni correction, suggesting a robust relationship with RFS. No effect of PD-L1 rs822336 was observed on OS (*p* = 0.564). Neither rs767649 nor rs1893650 miR-155/*MIR155HG* variants demonstrated significant associations with OS or RFS in our DLBCL cohort.

Significant variables in univariate models and those with a tendency (*p* < 0.100) were included in multivariate analysis, Table 5. For OS, IPI score (HR = 1.709, *p* = 0.006), PD-L1 rs4143815 (HR = 1.773, *p* = 0.044), and relapse status (HR = 2.575, *p* = 0.015) showed significant impact. Although rs4143815 entered intermediate multivariate steps, the association with OS did not meet the Bonferroni threshold in Cox analysis and was therefore interpreted as nominal. In the final step of the model, relapse status demonstrated the strongest association with OS and emerged as an independent predictor of OS in our DLBCL cohort (HR = 3.764, *p* = 0.0004).

For RFS, the multivariate model stepwise selection indicated a significant impact of IPI score (HR = 1.593, *p* = 0.022) and PD-L1 rs822336 (HR = 2.387, *p* = 0.003) on RFS, Table 5. In the final step of the model, PD-L1 rs822336 remained an independent predictor of RFS, with the GG conferring higher recurrence risk relative to the protective CC genotype (HR = 2.137, *p* = 0.011). When applying a variant-level Bonferroni-corrected significance threshold, the association of PD-L1 rs822336 with RFS remained statistically significant in multivariate Cox regression. In contrast, PD-L1 rs4143815 did not show a significant association with RFS.

### 3.5. Survival Analysis

**Figure 1:**
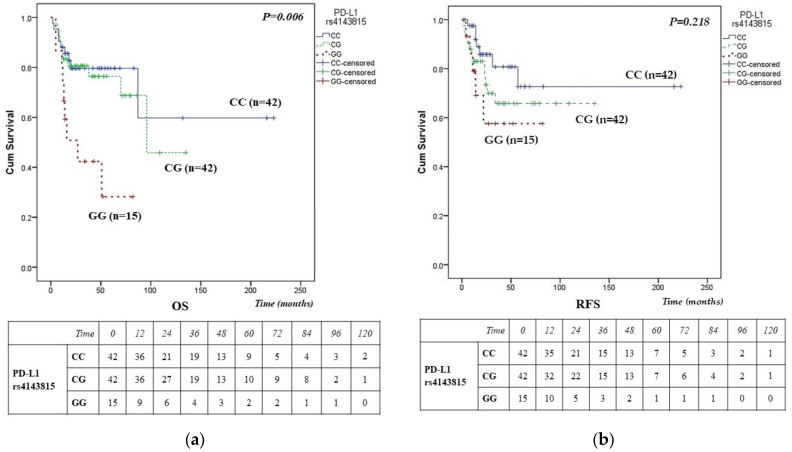
Kaplan–Meier survival curves of PD-L1/*CD274* gene variant rs4143815 (C>G) in DLBCL patients, assessed by the log-rank test. (**a**) Kaplan–Meier survival curves of OS (log-rank *p* = 0.006) (**b**) RFS for CC, CG, and GG genotypes of PD-L1 gene variant rs4143815 (log-rank *p* = 0.218). Numbers at risk displayed beneath the survival curves indicate patients alive and uncensored at the beginning of each time interval, while censored cases are excluded from subsequent time points.

During follow-up, 24 patients experienced relapse and 29 patients (29.3%) died. Median RFS was 24 months (range: 1–223), and median OS was 27 months (range: 2–223). The PD-L1 gene variant rs4143815 showed a significant association with survival, Figure 1. Patients carrying the variant homozygous genotype GG exhibited substantially reduced OS compared to the wt CC genotype, as demonstrated by Kaplan–Meier analysis (*p* = 0.006), Figure 1a. However, no association was observed between PD-L1 gene variant rs4143815 and RFS (*p* = 0.218), Figure 1b.

As shown in the Kaplan–Meier curves and the corresponding number-at-risk tables, Figure 1a, the rs4143815 GG genotype showed a rapid early decline in the number at risk for both OS and RFS within the 12–24 months period, whereas CC and CG genotypes demonstrated more gradual and parallel declines throughout follow-up. Beyond 36 months, survival estimates for the GG genotype should be interpreted with caution due to the reduced number of patients at risk.

**Figure 2:**
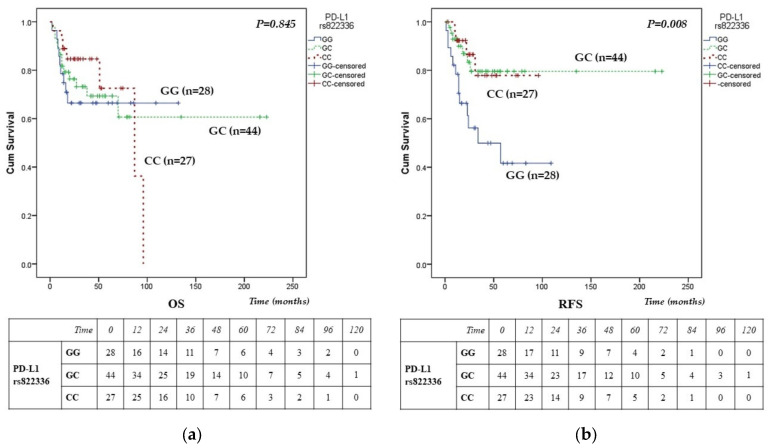
Kaplan–Meier survival curves of PD-L1**/***CD274* gene variant rs822336 (G>C) in DLBCL patients, assessed by the log-rank test. (**a**) Kaplan–Meier survival curves of OS (log-rank *p* = 0.845) (**b**) RFS for GG, GC, and CC genotypes of PD-L1 gene variant rs822336 (log-rank *p* = 0.008). Numbers at risk displayed beneath the survival curves indicate patients alive and uncensored at the beginning of each time interval, while censored cases are excluded from subsequent time points.

In contrast, the PD-L1 gene variant rs822336 showed the opposite pattern of association with OS and RFS, Figure 2. This variant was not associated with OS (*p* = 0.845), Figure 2a, but demonstrated a significant association with RFS (*p* = 0.008), Figure 2b. Interestingly, patients with the wt GG genotype had worse RFS, whereas carriers of the protective variant allele C exhibited a substantially reduced risk of recurrence, Figure 2b. These findings align with the multivariate Cox model, where PD-L1 rs822336 (GG) remained an independent predictor of worse RFS, Table 5. For PD-L1 rs822336, differences in the number at risk for OS between individual genotypes were not pronounced, Figure 2a. However, the rs822336 GG genotype exhibited early decline for RFS, with a marked reduction in the number at risk within the first 24 months compared with the GC and CC genotypes. Figure 2b.

Subgroup analyses revealed differential effects of the PD-L1 gene variant rs4143815 across clinical stratified groups. When stratified by cell-of-origin, the association between rs4143815 and OS was not significant in ABC-DLBCL (Kaplan–Meier, *p* = 0.562), whereas a statistically significant association was observed in the GCB subtype (*p* = 0.006). Age-stratified analysis further demonstrated that the rs4143815 variant was significantly associated with OS in patients older than 60 years (*p* = 0.043), while the trend did not reach statistical significance among patients ≤60 years (*p* = 0.083). When stratified by cell-of-origin, the association between rs822336 and RFS was borderline significant in ABC-DLBCL (Kaplan–Meier, *p* = 0.051), whereas no association was observed in the GCB subtype (*p* = 0.300). When stratified by therapy, among R-CHOP-treated patients (82/99, 82.8%), the association between PD-L1 rs4143815 and OS was borderline significant (Kaplan–Meier, *p* = 0.050), while a significant association between rs822336 and RFS was observed (Kaplan–Meier, *p* = 0.003). However, these subgroup analyses were underpowered due to limited sample sizes, especially those stratified by cell-of-origin, and should therefore be interpreted as hypothesis-generating.

### 3.6. Cancer Risk Analysis

The associations between the analyzed polymorphisms and DLBCL risk, summarized in Table 6, were assessed by comparing genotype distributions between the DLBCL cohort and healthy individuals, matched by age and sex. Although the variant GG genotype for PD-L1 rs4143815 was more frequent among DLBCL patients (15.2%) than controls (6.5%), this trend did not reach statistical significance after adjustment for age and sex (OR = 2.575, *p* = 0.062). Consistently, the recessive model approached but did not reach significance (OR = 1.606, *p* = 0.051). Additive and dominant genetic models did not show significant associations with DLBCL susceptibility. Genotype distributions did not differ significantly between cases and controls for PD-L1 rs822336 and *MIR155HG* rs1893650.

**Figure 3:**
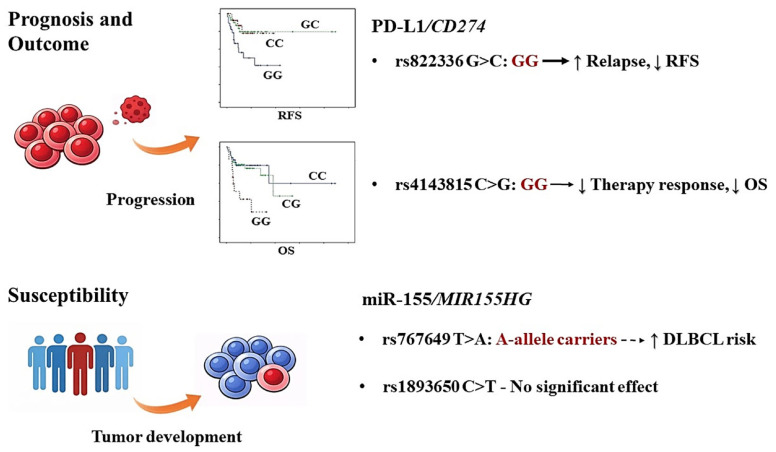
Schematic summary of key associations identified in this study. PD-L1/*CD274* variants were primarily associated with disease progression and survival outcomes. The rs822336 GG genotype was associated with increased relapse risk and shorter RFS and remained independently associated in multivariate Cox regression after variant-level Bonferroni correction. The rs4143815 GG genotype was associated with worse OS in Kaplan–Meier analysis, but did not retain significance after Bonferroni correction in Cox regression and was therefore considered nominal. The miR-155/*MIR155HG* rs767649 A-allele and AA genotype were nominally associated with increased susceptibility to DLBCL. Given the limited number of survival events and the low frequency of rare genotypes in susceptibility analyses, all illustrated associations should be considered hypothesis-generating and require validation in larger, independent cohorts.

In contrast, a notable association was observed for miR-155 rs767649. The rare AA genotype, although infrequent, was more prevalent among patients (7.1%) compared with controls (1.9%), corresponding to a more than five-fold increased risk (OR = 5.234, *p* = 0.045). The variant miR-155 rs767649 also showed significant association with DLBCL susceptibility under the additive model (*p* = 0.011) and the dominant model (*p* = 0.022), suggesting a potential dose-dependent effect of the variant A allele. However, these findings did not remain significant after Bonferroni correction, and, given the low frequency of the AA genotype and the limited statistical power for rare variants, these observations should be considered exploratory and require independent validation. Overall, the present data indicate that PD-L1/*CD274* variants are primarily associated with disease progression and survival outcomes, whereas the observed association for the miR-155 rs767649 represents an exploratory finding that warrants validation in larger, independent cohorts, Figure 3.

## 4. Discussion

Diffuse large B-cell lymphoma (DLBCL), the most frequent subtype of B-cell non-Hodgkin lymphoma (B-NHL), represents a biologically complex and clinically heterogeneous neoplasm whose course is shaped by genetic alterations, transcriptomic subtypes, and interactions with the immune microenvironment [1,2]. Despite significant advances in R-CHOP chemotherapy, a substantial proportion of patients still experience treatment failure, relapse, or refractory disease, underscoring the need for novel prognostic biomarkers and refined therapeutic strategies [4].

Germline variants in key immune-regulatory genes may modify RNA expression or stability, influencing the anti-tumor immune response, therapeutic response, and survival in DLBCL patients. Accordingly, this study examined selected variants in PD-L1-encoding gene *CD274* (rs4143815 and rs822336), and miR-155-encoding gene *MIR155HG* (rs1893650 and rs767649) in relation to susceptibility, clinicopathological features, and outcomes in DLBCL. Although functional validation was not performed in the present study, the investigated variants were selected based on prior evidence of their regulatory roles in immune signaling and cancer-related pathways. To our knowledge, this is the first study to investigate these specific genetic variants in DLBCL, and the first to examine them in lymphomas within the Serbian population.

Among the four preselected immunoregulatory variants, PD-L1 rs822336 demonstrated the most consistent association with relapse and RFS, remaining significant after variant-level Bonferroni correction and persisting as an independent predictor of RFS in multivariate analysis. PD-L1 rs4143815 additionally showed a significant association with OS in Kaplan–Meier analysis, but the multivariate Cox estimate did not meet the Bonferroni threshold, underscoring the need for validation in larger cohorts. Our findings also suggest the possible contribution of the miR-155 rs767649 variant to DLBCL susceptibility. However, given the very low frequency of the AA genotype in both cases and controls, this finding should be considered primarily as exploratory and warrants further validation in larger, multi-centric cohorts.

Our findings showing an association between PD-L1 rs822336 and relapse as well as RFS suggest that this variant may primarily influence early disease course rather than long-term mortality. Notably, the wild-type GG genotype was associated with worse RFS and remained an independent predictor in multivariate analysis, whereas the variant CC genotype exerted a protective effect. Such genotype-dependent differences may reflect context-dependent transcriptional regulation, whereby modest changes in PD-L1 expression shift the balance between effective anti-tumor immunity and immune escape. Multiple biological explanations remain possible, including linkage disequilibrium with other functional regulatory variants, population-specific genetic effects, and complex promoter-level regulatory dynamics. These findings should be interpreted in light of the moderate cohort size and limited number of events, which limit statistical power for genotype-stratified analyses. Accordingly, these observations are interpreted as biologically plausible but hypothesis-generating, and direct functional consequences cannot be inferred from the present genetic association data.

The divergence between PD-L1 rs822336 associations with RFS and OS may reflect specific biological mechanisms in different disease stages of DLBCL progression. Such dynamic regulatory effects are consistent with the complex immune-modulatory roles of PD-L1 in DLBCL and support the notion that rs822336 influences early clinical course without necessarily dictating overall survival. Relapse risk is often driven by minimal residual disease and the behavior of early tumor subclones within a permissive TME, which may be particularly sensitive to subtle changes in PD-L1 expression. In contrast, the absence of association with OS suggests that the initial impact of this variant may be mitigated by salvage therapy efficacy, treatment heterogeneity, or clonal evolution occurring in later stages, ultimately attenuating its long-term prognostic effect.

Bioinformatics profiling supports the functional relevance of the preselected candidate variants. Both PD-L1 genetic variants (rs4143815, rs822336) and the miR-155/MIR155HG variants (rs767649, rs1893650) are located within transcriptionally active regulatory regions enriched for enhancer or promoter marks. Although CADD and GERP scores predicted low deleteriousness, each SNP may alter transcription-factor motifs or miRNA-binding potential, causing subtle but biologically important regulatory effects. These germline variations may potentially be involved in fine-tuning of immune signaling pathways and contribute to heterogeneity in DLBCL susceptibility and outcomes.

PD-L1 rs822336 has been recognized as a functionally relevant promoter polymorphism that can modify the affinity of transcription factors for their binding motifs, alter promoter accessibility, and downstream PD-L1 transcriptional activity and protein expression. Experimental and bioinformatics analyses demonstrated that rs822336, located within the promoter region, can modulate the recruitment of key transcriptional activators, resulting in altered PD-L1 expression and contributing to poorer clinical outcomes in several tumor types [21,25,26,27]. Functionally, rs822336 is located in a regulatory promoter/enhancer element of PD-L1 and mediates allele-specific transcriptional responses through competitive binding of transcription factors C/EBPβ and NFIC. Their silencing differentially affected PD-L1 expression based on rs822336 genotype in human NSCLC cell lines, as well as their susceptibility to HLA class I–matched PBMCs incubated with anti-PD-1 monoclonal antibody nivolumab [28].

While there is no available data on PD-L1 rs4143815 and rs822336 variants in DLBCL and only limited data on B-NHL [21,22], our results are in line with the various studies, although the majority of the data derive from solid tumors. The variant CC genotype of PD-L1 rs822336 was reported to be an independent prognostic marker of a favorable prognosis in gastric cancer [27] and triple-negative breast cancer (TNBC) [20]. However, in hepatocellular carcinoma, the GG genotype has been associated with higher PD-L1 expression and poorer OS [25], while the C allele has been associated with reduced PD-L1 expression and poor survival in NSCLC [29]. Inconsistent effects of this genetic variant across multiple malignancies can be attributed to the context and discrepancies specific to cancer types.

Our findings that patients carrying the rs4143815 genotype GG had a significantly worse prognosis compared to CC genotype carriers, in line with the observed higher prevalence of the GG genotype among patients with inferior therapy response. As rs4143815 is localized in the 3′ UTR region of *CD274*, these findings are consistent with previously reported functional effects on PD-L1 expression and the consequent induction of a more immunosuppressive environment. The higher prevalence of the G allele, particularly the GG genotype, in treatment-resistant patients further supports its predictive potential. However, no statistically significant association of PD-L1 rs4143815 with relapse status or RFS was observed.

Variant rs4143815 has been associated with poor therapy response, low disease-free survival, and unfavorable outcomes in a variety of cancers [25,26,29,30,31,32,33,34]. Located in the 3′UTR of *CD274/B7-H1*, rs4143815 plays a significant regulatory role in PD-L1 gene expression. In non-small cell lung cancer (NSCLC), the luciferase assay demonstrated functional relevance of rs4143815, showing that the C allele was significantly associated with increased transcription activity compared with the G allele [29]. In addition, it was demonstrated that rs4143815 disrupts miR-570 binding, attenuates miRNA-mediated degradation, and thereby promotes PD-L1 upregulation and immune escape [35]. The functional effect was also demonstrated in pancreatic ductal adenocarcinoma, where serum PD-L1 levels were significantly lower in rs4143815 CC genotype carriers, increased in CG carriers, and highest in GG carriers, particularly in advanced disease stages. GG carriers also exhibited elevated serum tumor markers (CA19-9 and CEA), supporting a combined genotype- and stage-dependent association with aggressive tumor biology [26].

Stratified analyses by cell-of-origin (ABC or GCB-DLBCL) or age, although underpowered, suggested that the impact of PD-L1 variants may be context-dependent. For rs4143815, the association with OS was limited to the GCB subtype, implying a potentially stronger effect in tumors with lower baseline PD-L1 expression. For rs822336, a borderline association with RFS was observed in ABC-DLBCL but not in GCB cases. Age-stratification showed a similar pattern, with significance in patients older than 60 years, suggesting a potential interaction with immunosenescence. However, these subgroup analyses were underpowered and should be interpreted as exploratory.

In the present study, miR-155 rs767649 was primarily associated with DLBCL susceptibility rather than clinical outcome, suggesting a potential role at the level of tumor initiation rather than disease progression. Given the very low frequency of the AA genotype in both cases and controls, this association should be regarded as exploratory and requires validation in substantially larger, independent cohorts. A biologically plausible mechanism is supported by previously demonstrated functional effects of the A allele, which has been shown to modulate NF-κB binding affinity and influence miR-155 expression [36,37]. The divergent pattern observed, miR-155 variation influencing susceptibility, while PD-L1 variants affect disease course, supports distinct biological windows of action for these immune-regulatory loci.

### 4.1. Potential Clinical Implications and Future Research Directions

These findings primarily inform future research directions rather than immediate clinical application. Germline immunogenetic variants may contribute to inter-individual variability in treatment response, relapse risk, and survival in DLBCL. When considered alongside established clinical markers, host immune-regulatory variants may support hypothesis generation for improved prognostic modeling, depending on validation in larger cohorts.

The therapeutic landscape of DLBCL is rapidly evolving, particularly with the use of immune checkpoint inhibitors in relapsed or refractory disease. Host genetic profiling may provide additional information on the immune-related context. However, the present data do not support patient selection or treatment modification at this stage. Instead, germline PD-L1 variants could be explored as stratification variables in future biomarker-driven studies. Integrating gene variant data with PD-L1 protein expression assessed by immuno-histochemistry (IHC) could provide additional information, as genotype reflects constitutive expression capacity, whereas protein levels capture the actual TME state. Future studies should evaluate whether the incorporation of germline immunogenetic markers improves prognostic performance beyond established markers by providing insight into host immune response capacity. Larger, multi-center cohorts will be required to assess their added prognostic value and to explore potential interactions among immune-related loci, such as PD-L1, CTLA-4, PD-1, and HLA.

Future studies should include multi-center, ethnically diverse cohorts to enable adequately powered GWAS for discovering novel immune-related markers beyond candidate gene approaches. Multi-omics integration, combining germline variants with tumor transcriptomics, epigenetics, and proteomics, will be essential to elucidate how inherited variation modulates the DLBCL immune microenvironment. Functional studies, including CRISPR-edited lymphoma models, could dissect the mechanistic impact of immunogenetic variants on transcription factor binding, cytokine responsiveness, and immune evasion. Pharmacogenomic studies should also investigate whether immunogenetic variants modify ICI efficacy or toxicity.

Ultimately, the development of integrated risk models combining clinical, pathological, molecular, and germline genetic data represents a key objective in creating individualized treatment plans. Advancing precision medicine in DLBCL will require not only refined characterization of tumor biology but also a deeper understanding of the inherited immune landscape influencing disease behavior and therapeutic response.

### 4.2. Study Limitations

Our study has several limitations that should be acknowledged. The primary limitation is the relatively moderate cohort size, which may reduce statistical power, particularly in stratified analyses, and increases the possibility of false-negative or borderline associations. Given the candidate-gene design and the multiple statistical comparisons, the risk of type I error should be acknowledged, particularly for borderline *p*-values in the 0.04–0.06 range and subgroup analyses. The limited number of survival events, especially in stratified analyses and longer follow-ups, may reduce the precision of effect estimates, and nominal associations should be interpreted as hypothesis-generating. Based on Schoenfeld’s method, assuming 80% power and α = 0.05, the observed event numbers (29 deaths and 24 relapses) indicate that the study was primarily powered to detect relatively large effects (minimal detectable HRs were 4.27 for rs4143815 GG in OS and 3.51 for rs822336 GG in RFS). Formal testing of the proportional hazards assumption was not performed due to the limited number of events, which is acknowledged as a limitation of this study. Given the low frequency of the rare genotypes, observed associations with DLBCL susceptibility should be considered exploratory. In addition, the single-center design may limit generalizability. Accordingly, independent validation in larger, multi-center, and ethnically diverse DLBCL cohorts is required to confirm these findings and to minimize potential population-specific effects.

Functional validation of the investigated genetic variants was not performed, and PD-L1 protein expression could not be assessed by IHC, limiting direct correlation between germline variants and functional or protein-level effects. Future functional studies and PD-L1 IHC validation would be valuable to strengthen mechanistic interpretations of the observed associations.

Furthermore, the candidate-gene approach limits discovery, and genome-wide approaches or targeted sequencing may identify additional variants beyond those observed by the candidate-gene approach. Integrative assessment of haplotype analysis, linkage disequilibrium, and copy-number variation may provide further insights in future studies.

From a clinical perspective, although the DLBCL cohort was well-characterized, largely uniformly treated, and had long-term follow-up, some treatment heterogeneity (R-CHOP vs. R-EPOCH and salvage regimens) may have influenced associations with OS and RFS. Finally, as patients in this cohort did not receive immune checkpoint inhibitors, future studies incorporating immunotherapy response data may provide important pharmacogenomics insight and further enhance the clinical relevance of these findings.

## 5. Conclusions

These findings suggest, in a hypothesis-generating manner, that germline variants in the PD-L1-encoding gene *CD274* rs822336 and rs4143815 may influence disease progression and clinical outcome in DLBCL, while miR-155 variation rs767649 may contribute to disease susceptibility. The distinct association patterns, where the rs822336 wild-type GG genotype was associated with increased relapse risk and reduced RFS while the rs4143815 GG genotype was associated with worse OS, highlight the complex roles of immune regulatory pathways in lymphoma biology.

Given the central role of PD-L1 in immunosuppression and tumor immune escape, inherited variation within regulatory regions of *CD274* may represent a biologically plausible mechanism through which host genomic differences influence disease behavior and clinical outcomes. These findings support a potential role for germline immune genetics in prognostic assessment. As genotyping becomes increasingly accessible, the integration of genetic information with established clinical risk markers may help refine future risk stratification and influence personalized therapeutic strategies. Ultimately, understanding both tumor-intrinsic and host immune genetics will be essential for precise, risk-adapted management of aggressive B-cell lymphomas.

## Figures and Tables

**Table 1:** Demographic and clinicopathological characteristics of DLBCL cohort.

Variables		Total N	%
Sex	male	63	63.6
	female	36	36.4
Age > 60 years	≤60	59	59.6
	>60	40	40.4
Stage	I	5	5.1
	II	26	26.3
	III	21	21.2
	IV	47	47.5
ESR	<55.5	59	59.6
	>55.5	40	40.4
LDH	≤250	49	49.5
	>250	50	50.5
DLBCL Type	ABC	29	29.3
	GCB	24	24.2
	Unclassified	46	46.5
Therapy	R-CHOP	82	82.8
	PT based	6	6.1
	Hyper-CVAD/MA	7	7.1
	Other	4	4.0
Response to therapy	CR	73	73.7
	PR	8	8.1
	Refractory	18	18.2
ECOG performance status	No	54	54.5
	Yes (≥2)	45	45.5
Extranodal disease	0	60	60.6
	1	32	32.3
	2	6	6.1
	3	1	1.0
IPI Score	0	8	8.1
	1	22	22.2
	2	35	35.4
	3	27	27.3
	4	5	5.1
	5	5	2.0
ASCT	No	81	81.8
	Yes	18	18.2
FISH	BCL2−/c-MYC−	5	5.1
	BCL2+	12	12.1
	Double hit	9	9.1
	Triple hit	3	3.0
	cMYC+	2	2.0
Relapse status	negative	75	75.8
	positive	24	24.2

**Table 2:** In silico analysis of investigated PD-L1/CD274 (rs4143815, rs822336), and miR-155/MIR155HG (rs767649, rs1893650) variants.

	Variant	PD-L1/*CD274* rs4143815, C>G	PD-L1/*CD274* rs822336, G>C	miR-155/*MIR155HG* rs767649, T>A	miR-155/*MIR155HG* rs1893650, C>T
Feature /Database					
Locus/Gene		9p24.1, *CD274*	9p24.1, *CD274*	21q21, *MIR155HG*	21q21, *MIR155HG*
Genomic region		3′UTR miRNA binding site	Promoter	Upstream enhancer region of *MIR155HG*	Intronic region
1000Genomes		G = 0.7181, C = 0.2819	G = 0.6474, C = 0.3526	T = 0.852, A = 0.148	T = 0.5799, C = 0.4201
RegulomeDB score		0.55324, Moderate	0.55436, Moderate	0.99542, Strong	0.55436, Moderate
RegulomeDB Chromatin state/Cell type		Active enhancer, Strong transcription in immune cells	Active enhancer, active transcription in immune cells	Active TSS, enhancer, and transcription in immune cells	Active enhancer, Weak transcription in blood
HaploReg–Chromatin marks		RNA-binding marks	Promoter marks (H3K4me3, H3K27ac)	Enhancer marks (H3K4me1, H3K27ac)	Weak–moderate enhancer marks
HaploReg– motifs, Protein targets		miR-570, *FOXO1*, *FOXO3*, *IRF*, *Pax5*, *RXRA*, *SOX6*	*Gfi1*, *NRSF* *NFKB*, *BATF*, *EBF1*	*Evi-1*, *Osf2*, *GR*	*AP-2*, *Rad21*, *SMC3* *MEF2A*, *OCT2* *POL2*, *CFOS*
CADD/PHRED		1.081	1.621	2.941	6.846
GERP score		−0.60, Non-conserved	−2.06, Non-conserved	−3.65, Non-conserved	0.32, Weak
Main predicted functional effects *		G-allele → higher *PD-L1* expression	C-allele → lower *PD-L1* expression	Modulates miR-155 expression	Possible effect on miR-155 expression
		Higher *PD-L1* expression → poor survival	Higher *PD-L1* expression → poor survival	High miR-155 expression → inflammation	High MIR155HG → inflammation
Cancer-associated features		Cancer risk, therapy response, and survival	Survival in several cancer types	miR-155-driven inflammation, cancer risk	Regulatory variant, carcinoma risk

* All functional effects shown are derived from in silico bioinformatic predictions; in vitro validation was not performed.

**Table 3:** Associations of investigated genetic variants with clinicopathological characteristics of DLBCL cohort.

Variables		PD-L1/CD274						miR-155/MIR155HG					
		rs4143815 C/G			rs822336 G/C			rs767649 T/A			rs1893650 C/T		
		CC	CG	GG	GG	GC	CC	TT	TA	AA	CC	CT	TT
Sex	male	25	27	11	18	29	16	45	13	5	38	21	4
	female	17	15	4	10	15	11	25	9	2	19	16	1
	*p*	0.630			0.849			0.822			0.458		
Age	≤60	27	25	7	18	27	14	39	14	6	35	20	4
	>60	15	17	8	10	17	13	31	8	1	22	17	1
	*p*	0.490			0.611			0.277			0.493		
Stage	I/II	14	13	4	8	12	11	20	8	3	21	9	1
	III/IV	28	29	11	20	32	16	50	14	4	36	28	4
	*p*	0.890			0.461			0.625			0.378		
ESR	<55.5	23	28	8	18	25	16	41	14	4	35	21	3
	>55.5	19	14	7	10	19	11	29	8	3	22	16	2
	*p*	0.467			0.820			0.906			0.904		
LDH	≤250	21	21	7	13	21	15	37	11	1	31	17	1
	>250	21	21	8	15	23	12	33	11	6	26	20	4
	*p*	0.972			0.757			0.150			0.291		
DLBCL Subtype	ABC	14	10	5	10	15	4	24	3	2	15	12	2
	GCB	10	12	2	7	9	8	17	5	2	16	7	1
	Unclassified	18	20	8	11	20	15	29	14	3	26	18	2
	*p*	0.718			0.398			0.366			0.844		
Therapy response	CR	34	31	8	21	32	20	49	20	4	41	28	4
	PR	4	4	0	1	4	3	7	0	1	5	2	1
	Refractory	4	7	7	6	8	4	14	2	2	11	7	0
	*p*	0.026 *			0.842			0.263			0.686		
ECOG Performance status	Score < 2	10	12	2	7	9	8	15	7	2	16	7	1
	Score ≥ 2	32	37	13	25	33	24	46	25	11	44	35	3
	*p*	0.403			0.989			0.142			0.206		
Extranodal disease	No	20	24	10	15	24	15	35	16	3	34	19	1
	Yes	22	18	5	13	20	12	35	6	4	23	18	4
	*p*	0.167			0.064			0.963			0.574		
IPI Risk	Low (0–2)	31	26	8	17	30	18	44	16	5	39	22	4
	High (3–5)	11	16	7	11	14	9	26	6	2	18	15	1
	*p*	0.285			0.803			0.659			0.527		
ASCT	No	34	34	13	22	35	24	60	16	5	49	28	4
	Yes	8	8	2	6	9	3	10	6	2	8	9	1
	*p*	0.870			0.533			0.295			0.447		
Relapse status	No	35	30	10	15	37	23	52	17	6	45	26	4
	Yes	7	12	5	13	7	4	18	5	1	12	11	1
	*p*	0.299			0.005 **			0.784			0.615		

* Nominal statistical significance was defined as *p* < 0.05. ** Associations remaining significant after Bonferroni correction for four variants (*α* = 0.0125).

**Table 4:** Univariate analysis of different prognostic factors, according to Cox proportional hazards regression analysis for overall survival and recurrence-free survival.

Variables	OS		RFS	
	HR, [95% CI]	*p*	HR, [95% CI]	*p*
Age	1.003 [0.979–1.028]	0.795	1.015 [0.987–1.045]	0.300
Sex	0.525 [0.224–1.231]	0.138	1.162 [0.515–2.620]	0.718
Stage	1.078 [0.728–1.596]	0.709	1.258 [0.807–1.961]	0.310
ESR	1.209 [0.580–2.518]	0.613	0.980 [0.434–2.211]	0.961
LDH	1.449 [0.691–3.040]	0.327	1.489 [0.661–3.355]	0.337
DLBCL type	0.977 [0.627–1.522]	0.918	0.797 [0.480–1.322]	0.379
Therapy response	3.906 [2.545–5.988]	0.000001	1.650 [0.990–2.747]	0.055
ECOG status	2.121 [0.997–4.511]	0.051	1.220 [0.545–2.732]	0.629
Extranodal disease	0.698 [0.320–1.522]	0.366	0.774 [0.337–1.777]	0.545
IPI score (0–5)	1.932 [1.360–2.744]	0.0002	1.633 [1.111–2.399]	0.012
ASCT	0.645 [0.241–1.727]	0.383	1.451 [0.598–3.520]	0.410
Relapse status	3.764 [1.813–7.815]	0.0004	-	-
PD-L1 rs4143815 *	1.927 [1.146–3.242]	0.013	1.635 [0.933–2.865]	0.086
PD-L1 rs822336 *	1.156 [0.707–1.890]	0.564	2.137 [1.186–3.846]	0.011
miR-155 rs767649 *	0.921 [0.495–1.712]	0.794	0.733 [0.349–1.539]	0.412
MIR155HG rs1893650 *	1.142 [0.612–2.130]	0.676	1.162 [0.594–2.272]	0.661

* Cox models used the protective genotypes as the reference; HR—Hazard ratio. CI—Confidence interval.

**Table 5:** Multivariate analysis of different prognostic factors with overall survival and recurrence-free survival, according to Cox proportional hazards regression analysis.

	OS			RFS		
	Variable	HR [95% CI]	*p*	Variable	HR [95% CI]	*p*
Stepwise	IPI score (0–5)	1.709 [1.166–2.506]	0.006	IPI score (0–5)	1.593 [1.069–2.374]	0.022
	PD-L1 rs4143815 * GG	1.773 [1.016–3.095]	0.044	PD-L1 rs4143815 * GG	1.987 [1.101–3.584]	0.023
	Relapse status	2.575 [1.198–5.537]	0.015	PD-L1 rs822336 * GG	2.387 [1.339–4.266]	0.003
Independent	Relapse status	3.764 [1.813–7.815]	0.0004	PD-L1 rs822336 * GG	2.137 [1.186–3.846]	0.011

* Cox models used the protective genotypes CC for both variants as the reference; HR—Hazard ratio. CI—Confidence interval; Variables were entered into the multivariate models using stepwise forward selection based on univariate analysis (*p* < 0.100).

**Table 6:** Association of analyzed gene polymorphisms and DLBCL risk.

Gene/SNP	Genotype	Controls		DLBCL		Adjusted OR *, [95 CI]	*p*
		*n* = 113	%	*n* = 99	%		
PD-L1 rs4143815	CC	53	46.9	42	42.4	1 (Ref.)	-
	CG	53	46.9	42	42.4	0.998 [0.561–1.775]	0.994
	GG	7	6	15	15.2	2.575 [0.954–6.952]	0.062
	Additive model					1.341 [0.884–2.035]	0.168
	Recessive model—mt. vs. wt + ht (Ref.)					1.606 [0.998–2.582]	0.051
	Dominant model wt vs. het + mut (Ref.)					1.088 [0.828–1.430]	0.546
PD-L1 rs822336	GG	45	39.8	28	28.3	1 (Ref.)	-
	GC	42	37.1	44	44.4	1.630 [0.860–3.091]	0.134
	CC	26	23	27	27.3	1.625 [0.789–3.346]	0.188
	Additive model					1.292 [0.902–1.850]	0.162
	Recessive model—mt. vs. wt + ht (Ref.)					1.114 [0.814–1.524]	0.500
	Dominant model wt vs. het + mut (Ref.)					1.276 [0.953–1.708]	0.101
miR-155 rs767649	TT	94	83.2	70	70.7	1 (Ref.)	-
	TA	17	15	22	22.2	1.840 [0.899–3.765]	0.095
	AA	2	1.8	7	7.1	5.234 [1.039–26.355]	0.045
	Additive model					2.030 [1.173–3.513]	0.011
	Recessive model—mt. vs. wt + ht (Ref.)					2.149 [0.962–4.801]	0.062
	Dominant model—wt vs. het + mut (Ref.)					1.480 [1.059–2.066]	0.022
MIR155HG rs1893650	TT	58	51.3	57	57.6	1 (Ref.)	-
	TC	48	42.5	38	38.4	0.761 [0.431–1.344]	0.347
	CC	7	6.2	5	5.1	0.691 [0.204–2.340]	0.552
	Additive model					0.792 [0.502–1.251]	0.317
	Recessive model—mt. vs. wt + ht (Ref.)					0.881 [0.485–1.601]	0.678
	Dominant model—wt vs. het + mut (Ref.)					0.867 [0.659–1.141]	0.309

* Adjusted for age and sex. CI—confidence interval. Ref—reference genotype.

## Data Availability

The data presented in this study are available on request from the corresponding author due to privacy and ethical restrictions.

## Abbreviations

ABC	Activated B-cell-like
ASCT	Autologous stem cell transplantation
B-NHL	B-cell non-Hodgkin lymphoma
CD	Cluster of differentiation
CI	Confidence interval
CR	Complete response
CTL	Cytotoxic T lymphocyte
DLBCL	Diffuse large B-cell lymphoma
ECOG	Eastern Cooperative Oncology Group
ESR	Erythrocyte sedimentation rate
GCB	Germinal-center B-cell-like
GERP	Genomic Evolutionary Rate Profiling
GWAS	Genome-wide association study
HLA	Human leukocyte antigen
HR	Hazard ratio
ICI	Immune checkpoint inhibitor
IFN-γ	Interferon gamma
IHC	Immuno-histochemistry
IPI	International Prognostic Index
LDH	Lactate dehydrogenase
MHC	Major histocompatibility complex
miRNA	MicroRNA
*MIR155HG*	miR-155 host gene
ncRNA	Non-coding RNA
NK cells	Natural killer cells
NSCLC	Non-small cell lung cancer
OR	Odds ratio
OS	Overall survival
PBMCs	Peripheral blood mononuclear cells
PD-1	Programmed cell death protein 1
PD-L1	Programmed death ligand 1
PR	Partial response
qPCR	Quantitative polymerase chain reaction
R-CHOP	Rituximab, cyclophosphamide, doxorubicin, vincristine, prednisone
R-EPOCH	Rituximab, etoposide, prednisone, vincristine, cyclophosphamide, doxorubicin
RFS	Relapse-free survival
SNP	Single-nucleotide polymorphism
SNV	Single-nucleotide variant
SOCS1	Suppressor of cytokine signaling 1
TCR	T-cell receptor
TME	Tumor microenvironment
UTR	Untranslated region
wt	Wild-type
